# Defining a Critical Partition Zone for Sagittal Alignment in Lumbar Spine Fusion Surgery: A Systematic Review

**DOI:** 10.3390/bioengineering11121240

**Published:** 2024-12-08

**Authors:** Jie-Ren Mi Le, Wen-Tien Wu, Chih-Wei Chen, Fu-Shan Jaw, Shu-Hua Yang, Kuang-Ting Yeh

**Affiliations:** 1 Institute of Biomedical Engineering, College of Medicine and College of Engineering, National Taiwan University, Taipei City 106216, Taiwan; sylviafang01@gmail.com (J.-R.M.L.); jaw@ntu.edu.tw (F.-S.J.); 2 Department of Orthopedics, Hualien Tzu Chi Hospital, Buddhist Tzu Chi Medical Foundation, Hualien 970473, Taiwan; timwu@tzuchi.com.tw; 3 School of Medicine, Tzu Chi University, Hualien 970374, Taiwan; 4 Institute of Medical Sciences, Tzu Chi University, Hualien 970374, Taiwan; 5 Department of Orthopedics, National Taiwan University College of Medicine and National Taiwan University Hospital, Taipei 100225, Taiwan; ohw0701@gmail.com; 6 Graduate Institute of Clinical Pharmacy, Tzu Chi University, Hualien 970374, Taiwan

**Keywords:** sagittal alignment, lumbar spine, lumbar partitioning, biomechanical stability, spinal deformity surgery, pelvic incidence

## Abstract

Background: Sagittal alignment in the lumbar spine is essential for spinal stability and functionality, with significant implications in surgical planning for spinal deformity correction. However, standardized lumbar partitioning, particularly identifying a critical sagittal alignment zone, remains underdefined. This study aims to establish a reliable lumbar partition to guide surgical decisions and optimize clinical outcomes. Methods: A systematic review of four major biomedical databases yielded 32 studies, of which 4 met the inclusion criteria. Studies on asymptomatic adults with segmental lordosis data stratified by pelvic incidence were analyzed. Lumbar lordosis values were converted to percentages, allowing for cross-study comparison. Sensitivity analysis and bias assessment were performed to ensure methodological rigor. Results: The findings identified the L3–L5 interval, especially around the L4 vertebra, as a critical biomechanical zone across various populations and pelvic incidence groups. Individuals with higher pelvic incidence had concentrated lordosis in lower segments, while those with lower pelvic incidence had greater lordosis in upper segments, underscoring the L3–L5 region’s stability as a surgical reference. Conclusions: The L3–L5 interval serves as a key partition zone for sagittal alignment, providing a stable reference for lumbar spine fusion. These findings offer a foundational clinical reference, potentially improving alignment outcomes and reducing postoperative complications.

## 1. Introduction

The structural integrity and biomechanical functionality of the spine are essential for maintaining an upright posture, with the spine’s alignment significantly influencing physical health and quality of life. Sagittal alignment, particularly in the lumbar region, has a profound impact on the biomechanics of standing and movement, as it distributes loads and enables efficient energy use across spinal segments [1]. This alignment is often visualized as a natural S-curve, with deviations linked to conditions such as low-back pain [2,3], myelopathy [4], and decreased health-related quality of life [5,6]. As adult spinal deformities increasingly affect quality of life and physical performance, understanding sagittal alignment becomes crucial in surgical planning and outcomes [7,8]. Sagittal alignment in the lumbar spine helps preserve spinal function and provides mechanical stability by balancing the load between adjacent segments. It has been shown to vary significantly across populations due to factors such as ethnicity, age, and biomechanical differences [9,10,11,12]. Ethnicity, in particular, has been frequently associated with unique patterns of alignment that may contribute to different spinal pathologies; however, age, BMI, and lifestyle factors also significantly impact spinal curvature and warrant inclusion in alignment assessments to ensure a comprehensive understanding [12].

In recent years, segmental lordosis partitioning has gained importance in clinical and research settings, as distinct regions within the lumbar spine may have unique bio-mechanical properties influencing alignment [13,14,15]. The partitioning of the lumbar curve aids in identifying critical zones where the spine is most susceptible to biomechanical strain, degeneration, or injury [16,17,18]. Partitioning the lumbar spine is crucial for both understanding sagittal alignment and optimizing surgical planning, especially in procedures aimed at correcting spinal deformities or achieving stable fusion. The lumbar spine plays a central role in maintaining sagittal balance, distributing loads, and enabling efficient movement. However, variations in lordosis distribution across segments can result in uneven stress and lead to conditions such as adjacent segment degeneration, poor postoperative alignment, or junctional kyphosis if not properly addressed during surgery. For example, in lumbar spine fusion surgeries, accurately identifying critical zones of lordosis, such as the L3–L5 interval, can guide implant placement to minimize biomechanical strain on adjacent segments [19]. Similarly, in patients with adult spinal deformity, targeting specific lumbar partitions during corrective procedures helps achieve sagittal realignment and reduces compensatory mechanisms such as pelvic retroversion or hyperextension of the upper spine [20].

Laouissat et al. and Roussouly et al. have proposed partitioning methods for the lumbar spine based on biomechanical properties, with one approach defining the upper lumbar angle from the apex to the inflection point, while another emphasizes the role of the sacral slope [21,22]. The upper lumbar angle is relatively stable across populations, while the lower lumbar angle can vary significantly depending on pelvic incidence, illustrating the need for a standard segmentation approach that is applicable across diverse populations [21]. Pesenti et al. proposed an anatomical partition at the superior endplate of L4, defining proximal and distal lordosis segments that reflect biomechanical differences in the load-bearing characteristics of the lumbar spine [23]. Despite the utility of these methods, the current literature on lumbar partitioning remains fragmented, with each study contributing unique criteria for segmental analysis based on pelvic parameters. Our study seeks to consolidate and analyze these findings, identifying a critical partition zone that could serve as a standard reference for sagittal alignment. This zone is defined as the biomechanical transition region within the lumbar spine, playing a pivotal role in maintaining sagittal balance and optimizing load distribution. By refining and establishing specific boundaries for this critical partition zone, our study aims to provide a standardized framework that enhances the understanding of lumbar biomechanics and informs clinical decision-making. By examining the most frequently implicated regions in asymptomatic adults, particularly between the lower endplate of L3 and the upper endplate of L5, we aim to establish a reliable standard for lumbar segmentation that assists clinicians in preoperative planning and may reduce the risk of postoperative complications [23,24,25,26].

This review addresses a pressing gap in clinical practice by evaluating segmental lumbar lordosis data to provide a robust, biomechanically informed approach to spinal segmentation. The findings have the potential to improve outcomes in lumbar spine fusion surgery by identifying an optimal zone for partitioning, thus aligning clinical practices with biomechanical principles observed across populations [27,28,29].

## 2. Materials and Methods

### 2.1. Study Design and Registration

This study follows a systematic review and meta-analysis approach, registered in accordance with PROSPERO guidelines for methodological transparency. Registration in-formation and adherence to PROSPERO guidelines are included to enhance reproducibility (CRD42023493695).

### 2.2. Search Strategy

To conduct a comprehensive literature search, we accessed five major biomedical databases: PubMed, Scopus, Cochrane Library, EMBASE, and CINAHL. This search aimed to identify studies relevant to sagittal alignment partitioning in the lumbar spine. We utilized specific Boolean search terms, including “lumbar”, “lumbar lordosis”, “lumbosacral lordosis”, “segment”, “segmental lordosis”, “asymptomatic”, and “free of radiographic disease”. The search combined these terms using “AND” and “OR” operators to capture a broad yet relevant scope of studies. Our search spanned from inception to December 2023 to encompass all pertinent research on the topic.

### 2.3. Inclusion and Exclusion Criteria

To ensure the inclusion of high-quality studies, a rigorous screening process was implemented with the following predefined criteria:

1. Population: the studies involved asymptomatic adult participants to provide a baseline for lumbar alignment without confounding pathological factors.
2. Sample Size: The studies had a sample size of at least 100 participants, as larger cohorts yield more statistically reliable results. However, smaller studies were also considered where their inclusion added valuable data, with a risk of bias evaluation applied accordingly.
3. Measurement Parameters: the studies assessed lumbar sagittal alignment with quantifiable data on segmental lumbar lordosis, with stratification by pelvic incidence or sacral slope.
4. Data Accessibility: only studies with accessible full-text articles were included to allow for complete methodological evaluation.
5. Language: English-language studies only were included due to translation limitations and accessibility.

Studies were excluded if they involved symptomatic populations, did not report sufficient data on segmental lordosis, or lacked sample stratification by pelvic incidence or sacral slope.

### 2.4. Study Selection Process

The study selection process followed a PRISMA-based approach. Initially, two authors independently screened the titles and abstracts of the retrieved studies for relevance. Studies meeting the preliminary criteria underwent full-text review. Any discrepancies in the inclusion process were resolved through discussion with a third reviewer to ensure objective selection. The final selection comprised four studies that met all inclusion criteria.

### 2.5. Data Extraction

Data extraction was performed using a standardized form. For each study, the following variables were recorded: study design, population characteristics, sample size, pelvic incidence, segmental lordosis measurements, and specific lumbar partitions. Extracted lordosis data for each lumbar segment were converted into percentages where applicable, allowing for direct comparisons across studies. Each study’s risk of bias was assessed prior to data synthesis, following guidelines set by the ROBIS tool.

### 2.6. Statistical Analysis

#### 2.6.1. Conversion of Lordosis Values into Percentages

Segmental lordosis values from each study were converted into percentages relative to the total lumbar (L1–S1) curvature in degree and height in mm. This approach standardized the contribution of each segment to overall lordosis, allowing for cross-study comparison regardless of the original measurement units. For instance, the formula used defined 100% as the cumulative curvature from L1 to S1. Each segmental value was expressed as a proportion of this total curve, highlighting its relative contribution to the overall sagittal alignment.

#### 2.6.2. Stratification by Pelvic Incidence

Data from each study were stratified into pelvic incidence categories (e.g., low, mid, and high), as these classifications significantly influence lordosis distribution. This stratification ensured that the analysis accounted for variations in alignment across patient populations with differing anatomical and biomechanical characteristics.

#### 2.6.3. Trend Line Overlays

Segmental lordosis data from the included studies were plotted as trend lines using standardized percentages. Trend lines for each study were created using GraphPad Prism (version 6.0; GraphPad Software, San Diego, CA, USA). We overlaid these trend lines to identify intersection regions indicative of critical lumbar zones. Slope calculations between adjacent lordotic ratios enabled us to determine the transition regions, specifically focusing on the interval between the lower endplate of L3 and the upper endplate of L5, identified in previous research as a biomechanically significant region [30,31,32,33].

#### 2.6.4. Verification and Cumulative Analysis

The cumulative contribution of each segment to total lordosis was calculated and cross-verified with the original data. This ensured the accuracy of the standardization process and allowed for precise identification of the critical transition zone within the L3–L5 interval.

## 3. Results

### 3.1. Study Selection

**Figure 1:**
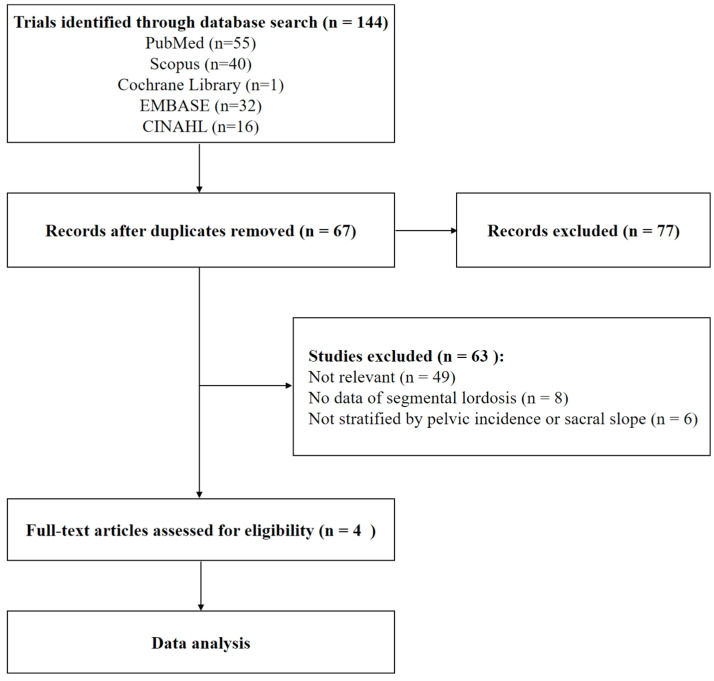
Flowchart of study selection.

A comprehensive database search was initially conducted to identify studies examining the intersection region of segmental lumbar lordosis in asymptomatic adults. A total of 144 studies were initially identified through the database search. After removing 77 duplicates, 67 studies remained for further screening. A stringent screening process, guided by predefined inclusion criteria, was employed to ensure analytical accuracy and reliability. Titles and abstracts were reviewed to determine relevance, and studies were excluded if they involved inappropriate study populations, insufficient sample sizes, or lacked adequate data on segmental lumbar lordosis. Following this screening process, 63 studies were excluded based on these criteria. Ultimately, only four studies met the inclusion criteria and were included in the final analysis. Figure 1 presents a flowchart of the study selection process for the literature review, detailing the number of studies assessed at each stage and the reasons for exclusions. This flowchart provides a transparent and reproducible overview of the selection process, enhancing the credibility and rigor of the literature review.

### 3.2. Study Characteristics

Table 1 provides an overview of the included studies, detailing sample size, study design, population characteristics, and methods of stratification by pelvic incidence. The studies included populations from various geographical regions—two from Western populations and two from Asia—allowing for a broad evaluation of sagittal alignment patterns across diverse ethnic groups [23,24,25,26].

The four studies used distinct approaches for lumbar spine partitioning:

1. Pesenti et al. divided the lumbar spine based on anatomical landmarks, setting the partition line at the superior endplate of L4 [23];
2. Chung et al. utilized Roussouly classification for stratification by pelvic incidence types [24];
3. Baker et al. applied a computed tomography-based approach to assess segmental lordosis in relation to pelvic incidence [25];
4. Mi Le et al. analyzed sagittal alignment across a comprehensive Taiwanese cohort, categorizing patients by pelvic incidence groups [26].

### 3.3. Lumbar Segmental Lordosis and Intersection Regions Based on Pelvic Incidence

This review analyzed four studies [23,24,25,26], each stratifying patients by pelvic incidence values to assess segmental lumbar lordosis. Measurements were converted to percentages to enable standardized comparisons across studies.

**Figure 2:**
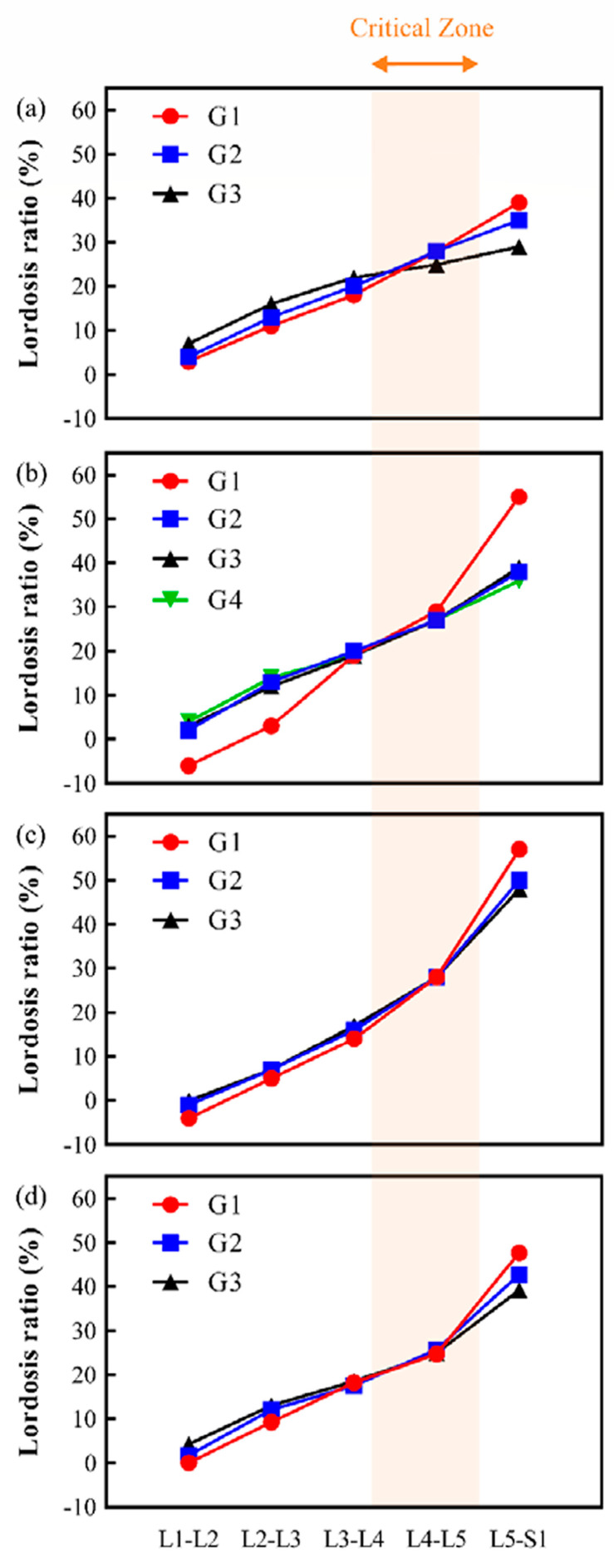
Critical zone (**a**) trends in the lordosis ratio from the data of Pesenti et al. [23]. Patients were divided into low- (<45°, G1), average- (45°−60°, G2), and high (>60°, G3)-PI groups. (**b**) Trends in the lordosis ratio from the data of Chung et al. [24]. Patients were grouped into type 1 (38.6° ± 7.9°, G1), type 2 (43.2° ± 6.3°, G2), type 3 (51.4° ± 6.8°, G3), and type 4 (62.9° ± 7.9°, G4). (**c**) Trends in the lordosis ratio from the data of Baker et al. [25]. Patients were categorized into low- (≤45°, G1), average- (46°−60°, G2), and high (>60°, G3)-PI groups. (**d**) Trends in the lordosis ratio from the data of Mi Le et al. [26]. Patients were stratified into low- (<45°, G1), average- (45°−55°, G2), and high (>55°, G3)-PI groups.

1. Pesenti et al. (United States) [23]: Patients were categorized into low- (< 45°), average- (45°–60°), and high (>60°)-pelvic-incidence groups, defined as G1, G2, and G3, as shown in Figure 2a. Intersection regions were identified within the L3–L5 segment for 40.4% to 72.7% of patients, while 0% to 21.9% had intersection regions in the L4–S1 segment (Table 1).
2. Chung et al. (Korea) [24]: Using the Roussouly classification, this study stratified patients into four pelvic incidence types: type 1 (38.6° ± 7.9°), type 2 (43.2° ± 6.3°), type 3 (51.4° ± 6.8°), and type 4 (62.9° ± 7.9°). The four groups were defined as G1, G2, G3, and G4, as shown in Figure 2b. Within these groups, intersection regions were observed between L3 and L5 in 34.1% to 57.1% of patients, with 9.8% to 24.2% showing intersection within L4–S1 (Table 2).
3. Baker et al. (New Zealand) [25]: Employing the same pelvic incidence groups as Pesenti et al., patients were divided into low (<45°), average (45°–60°), and high (>60°) categories. They were defined as G1, G2, and G3, as shown in Figure 2c. Intersection regions were observed within the L3–L5 segment for 83.6% to 100.0% of patients, while 0.0% to 50.6% showed intersection regions within the L4–S1 segment (Table 1). Notably, all patients displayed intersection regions exclusively within the L4–L5 segment, specifically between the upper endplate of L4 and the upper endplate of L5, with a lordosis ratio of 27.6% (Table 2).
4. Mi Le et al. (Taiwan) [26]: Patients were stratified into low- (<45°), average- (45°–55°), and high (>55°)-pelvic-incidence groups, defined as G1, G2, and G3, as shown in Figure 2d. In this study, 41.2% to 55.6% of patients exhibited intersection regions within the L3–L5 segment, while 2.3% to 24.2% had intersections in the L4–S1 region (Table 1 and Table 2).

Across all studies, we identified the upper and lower limits based on the findings of the four studies. These limits ranged from 34.1% to 100% at the L3–L5 interval and from 0% to 50.6% at the L4–S1 interval. This range was defined as the critical zone, as shown in Figure 2. In particular, these findings suggest that the L4 vertebra and the adjacent discs (L3/4 and L4/5 IDH) represent a critical biomechanical partition zone for lumbar sagittal alignment.

### 3.4. Vertebral and Intervertebral Disc Height Analysis

Zhang et al. [27] examined vertebral height (VH) and intervertebral disc height (IDH) within the lumbar spine, converting these values from millimeters to percentages for consistent comparison (Table 3). In the L3–L5 segment, the VH of L3 accounted for 33.8%, the IDH of the L3/4 disc was 15.2%, the VH of L4 represented 34.6%, and the IDH of the L4/5 disc comprised 16.4%. In the L4–S1 segment, the VH of L4 accounted for 34.3%, the IDH of L4/5 was 16.3%, the VH of L5 comprised 34.6%, and the IDH of L5/S1 was 14.8%.

## 4. Discussion

This study investigated the critical partition zone for sagittal alignment within the lumbar spine, identifying the L3–L5 interval as a key biomechanical region. Consistently across studies, this zone, especially the L4 vertebra and its adjacent intervertebral discs (L3/4 and L4/5), demonstrated potential as a reference point for lumbar partitioning in surgical planning. By consolidating data from multiple studies, we offer a comprehensive perspective on the biomechanical importance of this interval, which could support improved outcomes in lumbar spine fusion and other alignment-focused procedures.

### 4.1. Comparison with Existing Literature

The importance of sagittal alignment in spinal stability and functional outcomes is well supported in the literature. Proper alignment, particularly in the lumbar region, reduces compensatory mechanisms and enhances postoperative recovery and quality of life [5,6,8]. The findings of this study corroborate those of Laouissat et al. and Roussouly et al., who identified distinct biomechanical characteristics in upper and lower lumbar partitions influenced by pelvic incidence [21,22]. Specifically, the L4 vertebra was previously proposed as a transitional zone, supporting our finding that this vertebra and its surrounding segments serve as a natural dividing line in sagittal alignment [23]. This alignment variation across pelvic incidence groups, particularly evident in studies like Chung et al., indicates that sagittal alignment is influenced by unique patient factors [24]. Our review suggests that tailoring lumbar partitioning in surgical planning to these patient-specific characteristics could improve spinal stability and alignment, especially in populations with diverse pelvic parameters [24].

### 4.2. Clinical Implications of Lumbar Partitioning

**Figure 3:**
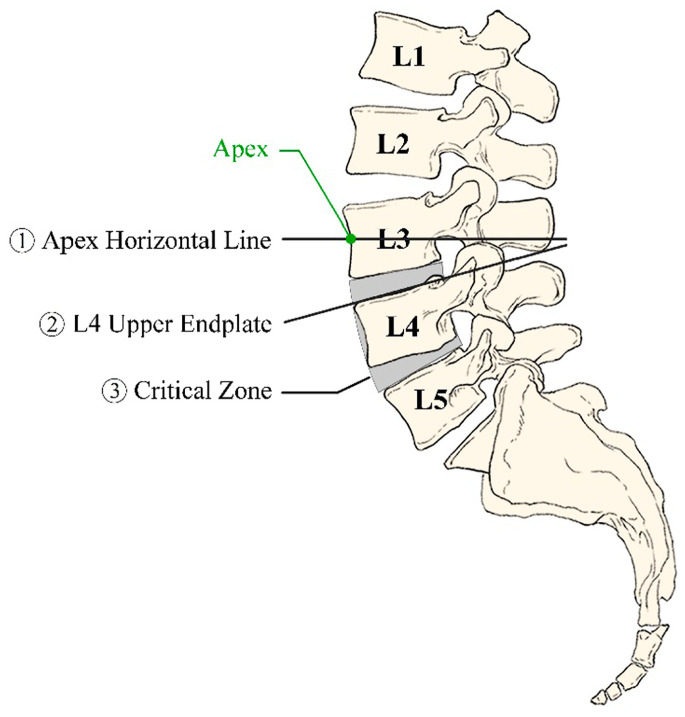
Partition position. (1) Apex horizontal line; (2) L4 upper endplate; (3) critical zone.

Identifying a critical lumbar partitioning zone has significant clinical implications, particularly for surgical planning and long-term patient outcomes in lumbar spine fusion (Figure 3). The consistent biomechanical significance of the L3–L5 interval across studies suggests that this region can help guide surgical targeting of specific lumbar segments to address sagittal imbalance effectively. Partitioning the lumbar spine based on this critical interval provides a stable reference that could reduce the risk of adjacent segment degeneration and postoperative complications, common challenges in spinal fusion [16,17,18]. This interval’s central role, especially the L4 vertebra, aligns with its recognized position as a biomechanical anchor within the lower lumbar curve, where it bears significant load, making it susceptible to degenerative changes [29,31,33]. Targeting the L3–L5 interval during surgical correction could help maintain alignment while respecting the spine’s natural load distribution, potentially minimizing complications like distal junctional kyphosis often seen with lower lumbar fusions [13,15]. Thus, the findings advocate a tailored approach that emphasizes this interval’s utility as a reference in lumbar spine partitioning, particularly in fusion surgeries where alignment and stability are paramount.

### 4.3. Limitations

While this review identifies a biomechanically significant partitioning zone, several limitations should be considered. The inclusion of only four studies may limit the generalizability of the findings. Although each included study had adequate sample sizes and standardized pelvic incidence stratification, methodological variability introduces potential bias. For example, the use of computed tomography by Baker et al., compared to the anatomical landmark approach of Pesenti et al., may lead to inconsistencies in measurements and interpretations of sagittal alignment [23,25]. Additionally, demographic and ethnic differences across study populations may influence lumbar alignment patterns, as spinal morphology varies by ethnicity [12]. While this review converted all measurements into standardized percentages for comparability, future research should examine the impact of demographic factors on the applicability of the L3–L5 interval as a universal partitioning zone. Furthermore, focusing solely on asymptomatic adults limits the clinical application of these findings to pathological conditions. Including symptomatic populations in future studies could help establish how degenerative changes alter the identified critical partition zone, adding a new layer of clinical relevance [9,18,29].

### 4.4. Methodological Considerations

This study adhered to PROSPERO registration and PRISMA guidelines to ensure methodological rigor and transparency. However, due to the limited number of studies, a formal meta-analysis was not feasible. Although trend lines and sensitivity analyses were used to identify the L3–L5 interval as a critical zone, an expanded dataset would enable statistical testing for a more robust evaluation. Larger-scale studies could allow for detailed analyses, including forest and funnel plots, to further validate this partitioning zone across varying conditions and populations [12,23]. Another methodological consideration was the conversion of segmental lordosis values to percentages, which standardized data across studies with different measurement units. Although this approach facilitated comparison, variability in how studies assess lordosis ratios remains a challenge. Future studies could benefit from a standardized protocol for lumbar sagittal alignment measurements, incorporating both anatomical and biomechanical markers to improve consistency and reliability [27].

### 4.5. Future Directions

Further investigation into the L3–L5 partition zone, especially in diverse populations, could refine surgical guidelines and enhance clinical outcomes. Research examining sagittal alignment variations within this interval across conditions like lumbar disc herniation, spinal stenosis, and spondylolisthesis would provide valuable insights [29,30,31]. Additionally, studies including symptomatic adults could evaluate how degenerative changes influence the L3–L5 zone’s biomechanical properties, potentially defining thresholds for surgical intervention based on alignment deviations [32,33]. Integrating advanced imaging techniques, such as dynamic MRI, could yield a more nuanced understanding of lumbar partitioning by evaluating segmental movement and load distribution within the critical zone in real time. This approach could clarify how alignment deviations contribute to mechanical stress and degeneration over time [21,34]. Future studies might also investigate the roles of muscle strength and pelvic tilt in maintaining sagittal balance, as these factors may compensate for or exacerbate segmental malalignment [10,14,15].

## 5. Conclusions

Our study establishes the L3–L5 interval, particularly around the L4 vertebra, as a critical partition zone for lumbar sagittal alignment. The consistent biomechanical significance of this interval across diverse populations suggests its potential as a standard reference for lumbar partitioning, especially in surgical planning for lumbar spine fusion and deformity correction. By aligning surgical approaches with these biomechanical insights, lumbar spine fusion outcomes may improve, with a potential reduction in postoperative complications and enhanced patient quality of life. While this study acknowledges limitations related to sample diversity and methodological variability, it provides a foundational reference that may inform future research and clinical applications. Further exploration into this partitioning approach could refine its utility, particularly in diverse patient populations and symptomatic cases, advancing the role of biomechanically informed practices in optimizing spinal alignment and patient outcomes in lumbar spine surgery.

## Figures and Tables

**Table 1:** Data transfer from millimeter to percentage.

Study	Stratification		L1–L2		L2–L3		L3–L4		L4–L5		L5–S1	
			Degree	Ratio	Degree	Ratio	Degree	Ratio	Degree	Ratio	Degree	Ratio
Pesenti (2018) [23]	Low PI	<45	1.4	2.7%	5.7	11.1%	9.4	18.3%	14.6	28.5%	20.2	39.4%
	Mid PI	45–60	2.3	3.9%	7.7	13.2%	11.6	19.9%	16.2	27.8%	20.5	35.2%
	High PI	>60	4.9	7.4%	10.8	16.4%	14.4	21.8%	16.5	25.0%	19.4	29.4%
Chung (2020) [24]	PI of Type 1	38.6 ± 7.9	−2.8	−6.1%	1.5	3.3%	8.5	18.6%	13.4	29.2%	25.2	54.5%
	PI of Type 2	43.2 ± 6.3	1.2	2.3%	6.5	12.6%	10.3	20.0%	13.7	26.5%	19.7	37.9%
	PI of Type 3	51.4 ± 6.8	1.8	3.1%	7.1	12.0%	11.3	19.1%	16	27.0%	22.7	38.1%
	PI of Type 4	62.9 ± 7.9	2.8	4.1%	9.4	13.8%	12.8	18.7%	18.7	27.3%	24.4	35.5%
Baker (2020) [25]	Low PI	≤45	−1.7	−4.2%	2.1	5.2%	5.7	14.2%	11.1	27.6%	23	57.2%
	Mid PI	46–60	−0.5	−1.0%	3.6	7.2%	7.9	15.8%	13.8	27.6%	25.2	50.4%
	High PI	>60	0	0.0%	4	7.4%	9.4	17.3%	15	27.6%	25.9	47.7%
Mi Le (2022) [26]	Low PI	<45	0.03	0.00	3.93	0.09	7.71	0.18	10.50	0.25	20.23	0.48
	Mid PI	45–55	0.94	0.02	6.17	0.12	8.91	0.18	13.13	0.26	21.76	0.43
	High PI	>55	2.48	0.04	7.50	0.13	10.67	0.18	14.44	0.25	22.62	0.39

**Table 2:** Intersection region of vertebrae height in percentage of patients.

Study	L3–L5	L4–S1
Pesenti (2018) [23] (*n* = 119)	40.4–72.7%	0.0–21.9%
Chung (2020) [24] (*n* = 160)	34.1–57.1%	9.8–24.2%
Baker (2020) [25] (*n* = 102)	83.6–100.0%	0.0–50.6%
Mi Le (2022) [26] (*n* = 324)	41.2–55.6%	2.3–16.7%

**Table 3:** Analysis results of vertebral height from data of Zhang (2018) [27].

Segment				mm	%	Cumulative %
L3–L5	L3–L4	L3	VH	23.1	33.8	33.8
		L3/4	IDH	10.4	15.2	49.0
	L4–L5	L4	VH	23.6	34.6	83.6
		L4/5	IDH	11.2	16.4	100.0
L4–S1	L4–L5	L4	VH	23.6	34.3	34.3
		L4/5	IDH	11.2	16.3	50.6
	L5–S1	L5	VH	23.8	34.6	85.2
		L5/S1	IDH	10.2	14.8	100.0

## Data Availability

There were no new data created in this study.
